# Development and Validation of [^3^H]OF-NB1 for Preclinical Assessment of GluN1/2B Candidate Drugs

**DOI:** 10.3390/ph15080960

**Published:** 2022-08-02

**Authors:** Hazem Ahmed, Livio Gisler, Nehal H. Elghazawy, Claudia Keller, Wolfgang Sippl, Steven H. Liang, Achi Haider, Simon M. Ametamey

**Affiliations:** 1 Center for Radiopharmaceutical Sciences ETH-PSI-USZ, Institute of Pharmaceutical Sciences ETH, Vladimir-Prelog-Weg 4, 8093 Zurich, Switzerland; hazem.farouk20@gmail.com (H.A.); liviogisler@hotmail.com (L.G.); claudia.keller@pharma.ethz.ch (C.K.); 2 Institute of Pharmacy, Department of Medicinal Chemistry, Martin-Luther-University Halle-Wittenberg, W.-Langenbeck-Str. 4, 06120 Halle, Germany; nehal.elghazawy@gmail.com (N.H.E.); wolfgang.sippl@pharmazie.uni-halle.de (W.S.); 3 Division of Nuclear Medicine and Molecular Imaging, Massachusetts General Hospital & Department of Radiology, Harvard Medical School, Boston, MA 02114, USA; liang.steven@mgh.harvard.edu

**Keywords:** GluN1/2B receptors, NMDA, [^3^H]ifenprodil, σ1 and σ2 receptors, receptor occupancy, PET imaging, drug development, neurodegenerative diseases

## Abstract

GluN2B-enriched *N*-methyl-D-aspartate receptors (NMDARs) are implicated in several neurodegenerative and psychiatric diseases, such as Alzheimer’s disease. No clinically valid GluN1/2B therapeutic exists due to a lack of selective GluN2B imaging tools, and the state-of-the-art [^3^H]ifenprodil shows poor selectivity in drug screening. To this end, we developed a tritium-labeled form of OF-NB1, a recently reported selective GluN1/2B positron emission tomography imaging (PET) agent, with a molar activity of 1.79 GBq/µmol. The performance of [^3^H]OF-NB1 and [^3^H]ifenprodil was compared through head-to-head competitive binding experiments, using the GluN1/2B ligand CP-101,606 and the sigma-1 receptor (σ1R) ligand SA-4503. Contrary to [^3^H]ifenprodil, the usage of [^3^H]OF-NB1 differentiated between GluN1/2B and σ1R binding components. These results were corroborated by observations from PET imaging experiments in Wistar rats using the σ1R radioligand [^18^F]fluspidine. To unravel the binding modes of OF-NB1 and ifenprodil in GluN1/2B and σ1Rs, we performed a retrospective in silico study using a molecular operating environment. OF-NB1 maintained similar interactions to GluN1/2B as ifenprodil, but only ifenprodil successfully fitted in the σ1R pocket, thereby explaining the high GluN1/2B selectivity of OF-NB1 compared to ifenprodil. We successfully showed in a proof-of-concept study the superiority of [^3^H]OF-NB1 over the gold standard [^3^H]ifenprodil in the screening of potential GluN1/2B drug candidates.

## 1. Introduction

*N*-methyl-D-aspartate receptors (NMDARs) are ionotropic excitatory neurotransmission mediators with pivotal functions in the central nervous system (CNS), particularly in learning and memory [1,2]. NMDARs are heterotetrameric constructs of GluN1/2/3 subunits, which are encoded by one, four and two genes, respectively [1,2]. The type of GluN2 subunit (A–D) dictates the function of the receptor and the unique spatial and temporal distribution across the brain [1,3,4,5,6]. In the adult brain, for instance, GluN1/2A is ubiquitously expressed, whereas the GluN1/2B is localized in the forebrain area [6,7,8]. Furthermore, the composition of NMDAR subunits varies depending on the localization within the neurons. While GluN1/2A is abundant at synaptic junctions, extrasynaptic NMDARs are particularly enriched with GluN1/2B [9]. Finally, the localization and function are closely linked; synaptic NMDARs promote neuronal survival upon stimulation, while extrasynaptic NMDARs are pro-apoptotic [9]. Notwithstanding the advances in our understanding of NMDARs, much remains to be elucidated with regards to the role of distinct subtypes in neuropathologies [4,10].

NMDARs are activated by the binding of the most abundant CNS excitatory neurotransmitter, glutamate, to the GluN2 subunit, while co-agonist glycine binds to the GluN1 subunit, thereby triggering a cascade of ion channel opening, Ca^2+^ influx and the subsequent signal transduction [3]. Analogous to their vital physiological functions, NMDARs play a key role in several neuropathologies, such as Alzheimer’s disease, Parkinson’s disease and stroke, and thus they have been the focus of drug development efforts for decades [9,11,12,13]. Initially developed as a competitive allosteric GluN1/2B antagonist, ifenprodil served as the template for multiple subunit-selective contemporary antagonists such as CP-101,606 and CERC-301 for the treatment of various neuropathologies [14]. The attention garnered toward GluN1/2B ligands stems from the detrimental role of the GluN1/2B receptors, in addition to the side effects that result from the attenuation of physiological NMDAR functions by non-subtype selective NMDAR-targeted ion channel blockers [15]. Accordingly, it is envisioned that subtype-selective GluN1/2B antagonism has the potential to elicit efficacy while being well tolerated. Nonetheless, the clinical development of GluN1/2B antagonists has been hampered by the lack of efficacy and significant off-target activity, thus necessitating more sophisticated drug development strategies to ensure appropriate GluN1/2B selectivity [14,16]. [^3^H]Ifenprodil is still hailed as the most commonly used radioligand for GluN1/2B competitive binding assays, despite the lack of selectivity over other CNS targets, especially regarding sigma receptors (K_i_ (σ1R) = 13 nM; K_i_ (σ2R) = 1.89 nM; K_i_ (GluN1/2B) = 11 nM) [17,18]. Research efforts to characterize the binding of [^3^H]ifenprodil shed light on the effect of low temperatures of 4 °C on the preferential binding of ifenprodil toward NMDARs [19]. However, performing binding affinity assays at 4 °C is not ideal, and could influence the behavior of the ligands under investigation. In light of this information, the search for a highly selective tritiated GluN1/2B antagonist is vital. In addition to the competitive receptor binding assays, a suitable tritiated ligand can be used in autoradiography experiments and saturation assays to determine the receptor expression (B_max_) levels.

**Figure 1:**
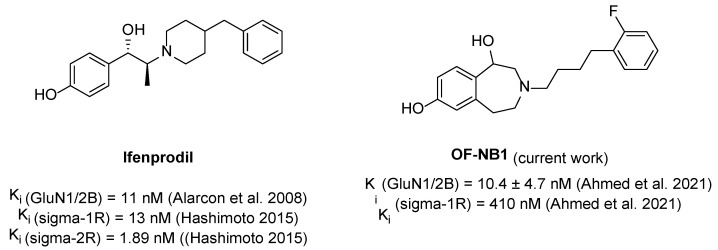
Left: Structure of ifenprodil, first ligand described for binding to the ifenprodil binding site of the GluN1/2B. Right: Structure of OF-NB1, a selective GluN1/2B ligand that is investigated in the current work.

Positron-emission tomography (PET) has become a vital tool in early drug development and has been used effectively to determine drug uptake, distribution, target engagement and receptor occupancy in vivo [20,21]. Previously published data suggested that 2,3,4,5-tetrahydro-1H-3-benzazepines constitute promising GluN1/2B ligands with regard to selectivity and potency [22,23,24,25,26,27,28,29]. A particularly auspicious ligand from this series is OF-NB1, which exhibits high GluN1/2B affinity and high selectivity over σ1Rs (Figure 1, K_i_ (GluN1/2B) = 10.4 ± 4.7 nM; K_i_ (σ1R) = 410 nM) [22]. This ligand was radiofluorinated with fluorine-18 for PET imaging studies and showed excellent accumulation in GluN1/2B-rich brain regions, namely the cortex, hippocampus, thalamus and striatum. Furthermore, it exhibited excellent specificity and selectivity when challenged with GluN1/2B and σ1R ligands. With regards to its physicochemical properties, OF-NB1 exhibited a log *D*_7.4_ of 2.05 ± 0.08 (n = 4), which is optimal for brain penetration [29]. As such, we tritiated OF-NB1 with the ultimate goal of providing a highly selective probe to facilitate GluN1/2B-targeted drug development. To support this goal, blocking studies with the GluN1/2B antagonist CP-101,606 were conducted using the σ1R PET tracer, [^18^F]fluspidine, in Wistar rats in vivo [30]. Subsequently, we performed docking studies of OF-NB1 and ifenprodil in order to substantiate the experimental results.

## 2. Results and Discussion

**Scheme 1:**
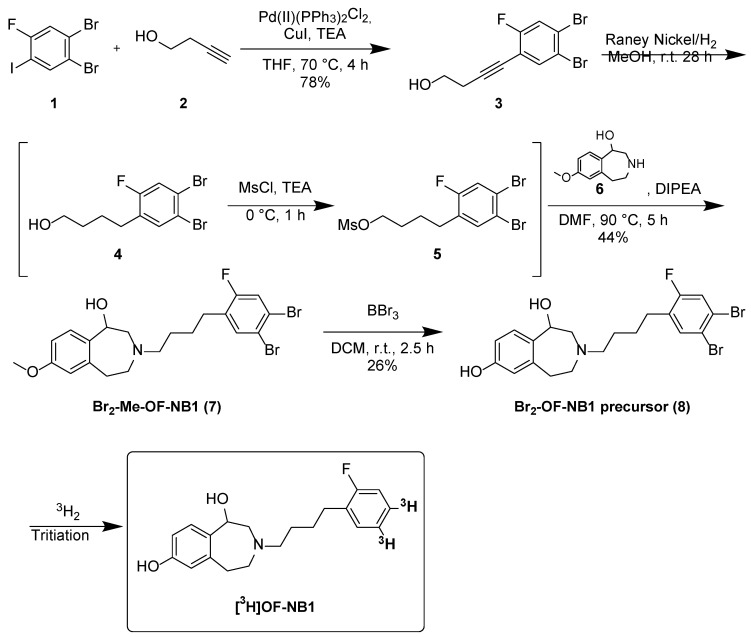
Synthetic pathway toward dibromo precursor **8** and [^3^H]OF-NB1.

At the outset of the studies, a five-step synthetic route was devised to prepare the dibromine-bearing precursor for the radiosynthesis of [^3^H]OF-NB1 (**7**) (Scheme 1). Sonogashira coupling of substituted iodobenzene **1** and alkyne **2** yielded benzalkyne alcohol **3** in 78% yield. Alcohol **3** was reduced with Raney nickel, subsequently treated with MsCl, and the resulting intermediate was used for the N-alkylation of commercially available bezazepine derivative **6** to give the N-alkylated product **7**, with a yield of 44% over three steps. The final demethylation of **7** using BBr_3_ gave the desired precursor **8** with a yield of 26%. Tritium-labeling of precursor **8** yielded [^3^H]OF-NB1 with 99% radiochemical purity and a molar activity of 1.79 GBq/µmol (48.3 Ci/mmol).

**Figure 2:**
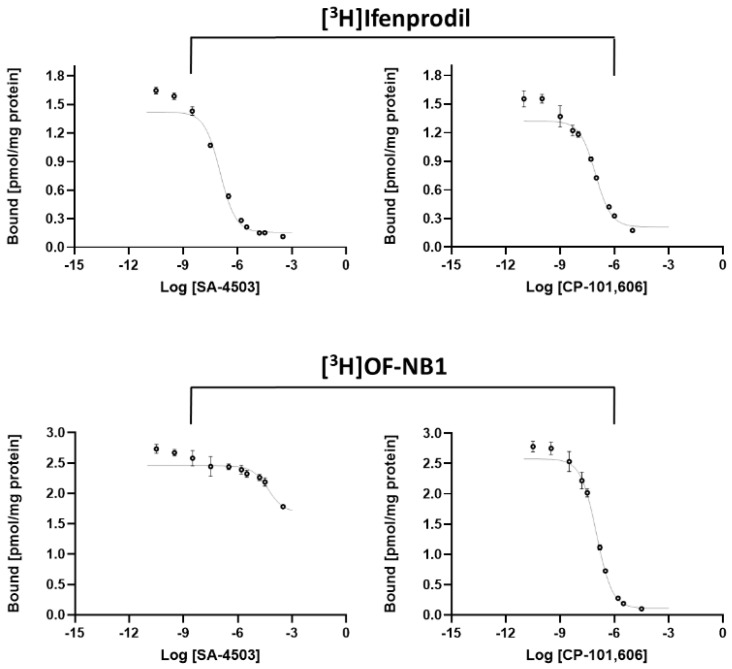
Representative saturation binding curves of the σ1R ligand SA-4503 (left) and the GluN1/2B antagonist CP-101,606 (right) using the two radioligands [^3^H]ifenprodil and [^3^H]OF-NB1.

In order to evaluate the viability and superiority of [^3^H]OF-NB1 relative to the commercially available [^3^H]ifenprodil, we determined the K_i_ (GluN1/2B) values of the GluN1/2B antagonist CP-101,606 and the σ1R ligand SA-4503 using both tritiated radioligands (Figure 2). Furthermore, we assessed the selectivity of the two drugs over σ1Rs using the σ1R radioligand, (+)-[^3^H]pentazocine. The results are summarized in Table 1.

The results revealed a two-digit nanomolar GluN1/2B affinity for CP-101,606 when using either of the two radioligands, [^3^H]OF-NB1 or [^3^H]ifenprodil. Both values were, however, considerably higher than the reported literature value of 16 nM [31]. Three notable differences comparing the assay at hand from the one described in literature, are as follows: (1) the absence of other agents that block non-NMDA receptors; (2) the usage of whole rat brain homogenates; and (3) the higher temperature of 25 °C.

The superiority of [^3^H]OF-NB1 over [^3^H]ifenprodil was evidently established when testing the σ1R ligand SA-4503. The K_i_ (GluN1/2B) value exceeded 100 µM (Table 1) when using [^3^H]OF-NB1 compared to 51 nM (Table 1) when using [^3^H]ifenprodil. The key reason for such a disparity is that SA-4503 is an extremely potent σ1R-selective ligand; however, ifenprodil binds to both GluN1/2B and σ1Rs indiscriminately. The K_i_ (σ1R) value of 3.8 nM for SA-4503 matched the published value of 4.6 nM [32]. On the other hand, the K_i_ (σ1R) value of 94 nM for CP-101,606 was considerably close to the published value of 60 nM using the σR ligand, [^3^H](3-(3-hydroxyphenyl)N-(1-propyl)-piperidine (3-PPP) [33]. This shows that CP-101,606 potentially exhibits a σ1R binding component, thereby providing a valid explanation for the higher K_i_ (GluN1/2B) value of CP-101,606 when using [^3^H]OF-NB1, given its low σ1R affinity as opposed to ifenprodil.

**Figure 3:**
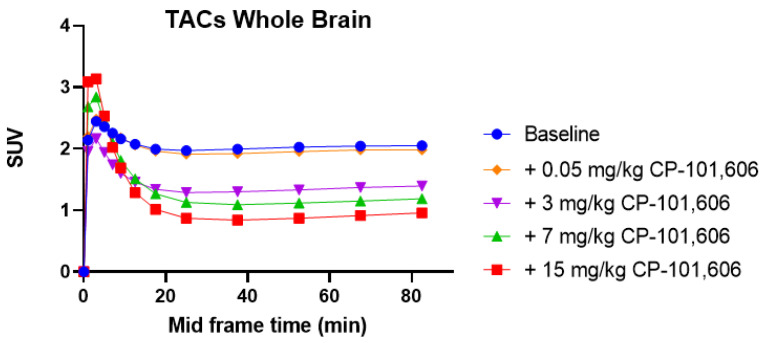
Time activity curves (TACs) of Wistar rat brain uptake of the σ1R tracer [^18^F]fluspidine under baseline and blockade conditions using the GluN1/2B antagonist CP-101,606 (0.05, 3, 7 and 15 mg/kg). Standard uptake values (SUVs) are averaged from 0–90 min.

**Figure 4:**
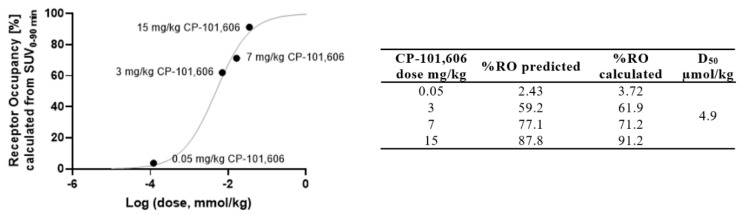
In vivo receptor occupancy (RO) of GluN1/2B antagonist CP-101,606 in Wistar rats in the presence of the σ1R tracer [^18^F]fluspidine. Plotting the calculated receptor occupancy against the different dose regimes allowed the calculation of D_50_ which is the administered dose of CP-101,606 that occupies 50% of the σ1Rs [23].

To verify our findings of CP-101,606 possessing significant σ1R binding, we conducted in vivo PET imaging studies with [^18^F]fluspidine, a σ1R radioligand which is known for its lack of uptake in the brain of σ1R knock-out mice [22,30]. These findings are depicted in Figure 3 and Figure 4.

In vivo PET results pertaining to the off-target characteristics of CP-101,606 toward σ1Rs corroborated the results obtained from the in vitro binding study. A series of doses were investigated, ranging from 0.05–15 mg/kg. Importantly, the GluN1/2B ligand, CP-101,606, displayed a dose-dependent blockade similar to that of the σ1R ligand SA-4503 [22]. This allowed us to calculate the D_50_ (σ1R), which indicates the administered dose of CP-101,606 that occupies 50% of the σ1Rs (Figure 3). The D_50_ (σ1R) of CP-101,606 was calculated to be 4.9 µmol/kg, which is higher than the D_50_ (GluN1/2B) of 8.1 µmol/kg [26]. This strong σ1R binding behavior of CP-101,606 offsets its unwarranted reputation of being a selective GluN1/2B. There have been efforts to develop ligands with dual GluN1/2B and σ1R activity [34]. Nonetheless, for the purpose of developing selective GluN1/2B ligands, one has to consider fundamentally reformulating the development strategy.

**Figure 5:**
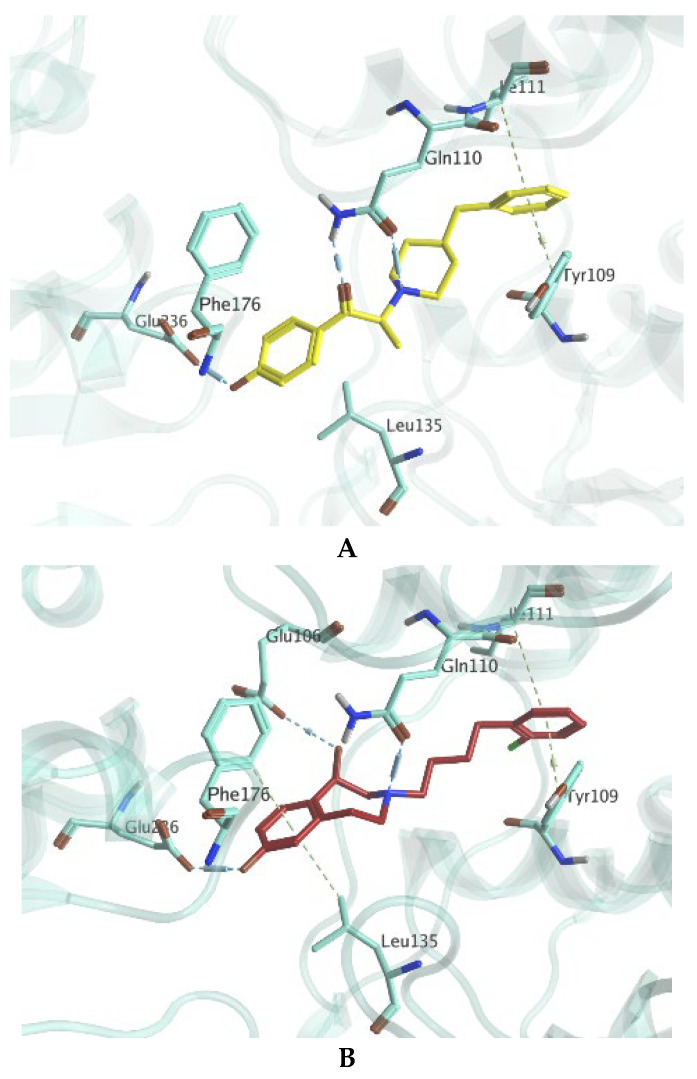
Predicted binding modes and possible interactions with GluN1/2B of ifenprodil (**A**) and OF-NB1 (**B**). Ifenprodil and OF-NB1 are depicted in yellow and maroon, respectively.

With the aim to better understand the interactions of OF-NB1 on a molecular level, the binding mode was studied alongside the two target receptors, GluN1/2B and σ1Rs, in a retrospective in silico study. With regards to GluN1/2B, the binding of OF-NB1 was compared to ifenprodil, which was previously co-crystallized with GluN1/2B (PDB-ID: 3QEL). As shown in Figure 5A, the binding of ifenprodil can be credited to the existence of three H-bonds with Gln110 and Glu236. Additionally, the two phenyl rings of ifenprodil guided multiple hydrophobic interactions with amino acids from the two GluN subunits, such as Tyr109 (GluN1A) and Ile111 (GluN2B). While OF-NB1 was able to maintain common interactions as ifenprodil as shown in Figure 5B, it surprisingly managed to form two additional interactions. Specifically, it should be noted that OF-NB1 achieved a new H-bond with Glu106 via its secondary hydroxyl functionality. Furthermore, the distant phenyl ring of OF-NB1 makes van-der-Waals interactions with amino acids Phe176 and Leu135. These additional interactions are energetically favorable, thus contributing positively to the binding affinity of OF-NB1 toward GluN1/2B.

**Figure 6:**
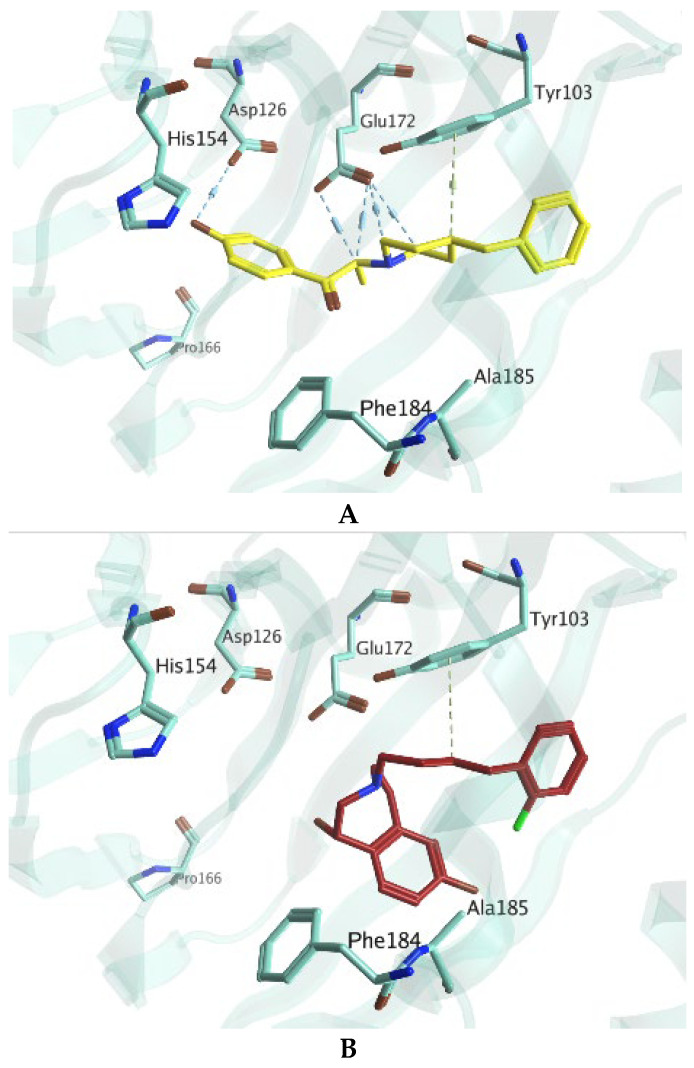
Predicted binding modes and possible interactions with σ1R of ifenprodil (**A**) and OF-NB1 (**B**). Ifenprodil and OF-NB1 are depicted in yellow and maroon, respectively.

The co-crystal structure of σ1R with pentazocine was used (PDB-ID: 6DK1) for the docking of ifenprodil and OF-NB1 against σ1R. Both Schmidt et al. and the Glennon model for σ1R ligands highlighted the prominence of the presence of a positively charged nitrogen in all σ1R ligands, where it forms a salt-bridge with Glu172 [35]. Such an interaction was noted in the docking of ifenprodil, in addition to further interactions with Tyr103 and Asp126 (Figure 6A). Conversely, OF-NB1 was only able to maintain van-der-Waals interaction with Tyr103 and even failed to interact with the crucial Glu172, despite its positively charged nitrogen (Figure 6B). Such behavior could be explained by the odd orientation exhibited by OF-NB1 within the σ1R binding pocket. The failure of OF-NB1 to align with ifenprodil supports the observations from the in vitro saturation binding experiments.

## 3. Materials and Methods

### 3.1. General Methods

All non-aqueous reactions were performed under N_2_ atmosphere using flame-dried glassware and standard syringe/septa methods unless stated otherwise. Reactions were magnetically stirred and further monitored by thin layer chromatography (TLC) performed on Merck TLC aluminum sheets (silica gel 60 F254). TLC spots were visualized using UV light (λ = 254 nm) or through staining with KMnO_4_ solution. Chromatographic purification of products was performed using SiliaFlash P60 silica gel (Silicycle) for preparative column chromatography with a particle size of 40–63 μm (230–400 mesh). Reactions at −78 °C were cooled in a dry ice/acetone bath and reactions at 0 °C were cooled in an ice/water bath. Chemicals were purchased from ABCR, Acros Organics, Amatek Chemical, Fluorochem, Merck, Perkin Elmer and Sigma Aldrich and used without further purification. Solvents for TLC, extraction and flash column chromatography were of technical grade. Extra dry solvents for all non-aqueous reactions were supplied by Acros Organics (puriss., dried over molecular sieves, water content <0.005%). Deuterated solvents (D, 99.9%) were purchased from Cambridge Isotope Laboratories, Inc. NMR spectra of compounds 3, 7 and 8 are presented in the Supplementary Material (Section S1). Tritium labeling was performed by RC Tritec AG (Teufen, Switzerland). Quality control chromatogram and mass spectrum are presented in the Supplementary Material (Section S2, Figures S1 and S2). Positron emission tomography (PET) imaging was performed in Wistar rats according to a previously published procedure [22]. PET images are presented in the Supplementary Materials (Section S3, Figure S3).

### 3.2. Mass Spectrometry

High-resolution mass spectra were obtained by the mass spectrometry service of the ETH Zürich Laboratorium für Organische Chemie on a Varian IonSpec FT-ICR (ESI), a Bruker Daltonics maXis ESI- QTOF spectrometer (ESI), a Bruker Daltonics SOLARIX spectrometer (MALDI), or a Bruker Daltonics UltraFlex II spectrometer (MALDI-TOF). For ESI (+MS) an enhanced quadratic calibration mode was used with the following reference mass peaks: 118.0863, 322.0481, 622.0290, 922.0098, 1221.9906, 1521.9715, 1821.9523, 2121.9332, 2421.9140, and 2721.8948.

### 3.3. NMR Spectroscopy

^1^H, ^19^F and ^13^C nuclear magnetic resonance (NMR) were recorded at room temperature on a Bruker Avance FT-NMR (400 MHz) with CDCl_3_ or methanol-*d*_4_ as solvent. Chemical shifts values (δ) are reported in parts per million (ppm) relative to tetramethylsilane (0.00 ppm) as an internal standard and the appropriate CDCl_3_ solvent signals (δ_H_ = 7.26 ppm and δ_C_ = 77.16). For ^1^H NMR spectra, resonance multiplicities are abbreviated as s = singlet, d = doublet, t = triplet, m = multiplet. Coupling constants (*J*) are reported in hertz (Hz).

### 3.4. Synthetic Procedures

#### 3.4.1. 4-(4,5-Dibromo-2-fluorophenyl)but-3yn-1-ol (**3**)

To a solution of 1,2-dibromo-4-fluoro-5-iodobenzene (1.0 eq., 1.58 g, 4.15 mmol) in THF (11.9 mL), but-3-yn-1-ol (1.2 eq., 0.38 mL, 4.98 mmol) and copper(I) iodide (0.2 eq., 0.16 g, 0.83 mmol) were added. To the stirring mixture, triethylamine (5 eq., 2.9 mL, 20.75 mmol) was added dropwise and left to stir for 15 min. Bis(triphenylphosphine)palladium(II)dichloride (0.1 eq., 0.33 g, 0.47 mmol) was added, and the mixture was refluxed for 4 h. After cooling to room temperature, the mixture was diluted with EtOAc (100 mL) and filtered through Celite using EtOAc (2 × 200 mL) as a solvent. The filtrate was concentrated under reduced pressure, and the resulting oily crude was purified by flash column chromatography (gradient elution with hexane:EtOAc, 90:10 to 70:30) to afford the title compound **3** (1.34 g, 4.15 mmol, 78%) as a white solid. ^1^H NMR (400 MHz, CDCl_3_) δ 7.65 (d, *J* = 6.9 Hz, 1H), 7.36 (d, *J* = 8.5 Hz, 1H), 3.83 (t, *J* = 6.3 Hz, 2H), 2.71 (t, *J* = 6.3 Hz, 2H), 1.84 (s, 1H). ^13^C NMR (101 MHz, CDCl_3_) δ 161.4 (d, *J* = 255.8 Hz), 137.3, 124.8 (d, *J* = 9.5 Hz), 121.0 (d, *J* = 26.1 Hz), 119.4, 113.2 (d, *J* = 17.4 Hz), 94.7, 73.9, 61.0, 24.1. ^19^F NMR (376 MHz, CDCl_3_) δ −110.8. HRMS (EI) calcd for C_10_H_7_OBr_2_F [M] 319.8848; found 319.8841.

#### 3.4.2. 4-(4,5-Dibromo-2-fluorophenyl)butan-1-ol (**4**)

To a stirring solution of 4-(4,5-dibromo-2-fluorophenyl)but-3yn-1-ol (1.0 eq., 1.04 g, 3.23 mmol) in MeOH (30 mL), Raney nickel (436 µL, 3.76 mmol) was added under nitrogen atmosphere. The reaction mixture was hydrogenated at atmospheric pressure for 26 h. The mixture was filtered through a filter paper using EtOAc (3 × 20 mL) as a solvent. The filtrate was concentrated under reduced pressure and the resulting oily crude (1.05 g, 3.22 mmol) was used in the next reaction without further purification.

#### 3.4.3. 4-(4,5-Dibromo-2-fluorophenyl)butan-1-ol (**5**)

To a stirring solution of 4-(4,5-dibromo-2-fluorophenyl)butan-1-ol (1 eq., 1.05 g, 3.22 mmol) in DCM (33 mL), triethylamine (1.8 eq., 0.8 mL, 5.79 mmol) was added dropwise. The mixture was cooled to 0 °C and methanesulfonyl chloride (1.0 eq., 0.25 mL, 3.22 mmol) was added dropwise. The solution was allowed to come to room temperature and was stirred for 1.5 h. The mixture was quenched with water (250 mL) and extracted with DCM (3 × 250 mL). The combined organic layers were washed with brine (300 mL), dried over MgSO_4_, filtered and concentrated under reduced pressure affording 4-(4,5-dibromo-2-fluorophenyl)butyl methanesulfonate (1.3 g, 3.22 mmol) as a yellow oily crude, which was used in the next reaction without further purification.

#### 3.4.4. 3-(4-(4,5-Dibromo-2-fluorophenyl)butyl)-7-methoxy-2,3,4,5-tetrahydro-1h-benzo[d]azepin-1-ol (**7**)

To a stirring solution of 7-methoxy-2,3,4,5-tetrahydro-1H-benzo[d]azepin-1-ol 6 (1.0 eq., 0.519 g, 2.69 mmol) and 4-(4,5-dibromo-2-fluorophenyl)butyl methanesulfonate (1.2 eq. 1.3 g, 3.22 mmol) in DMF (17.3 mL), DIPEA (0.87 eq., 0.4 mL, 2.35 mmol) was added dropwise. The mixture was refluxed for 5 h. After cooling the solution to r.t., it was quenched with aq. NaOH (200 mL, 1 mM) and extracted with DCM (3 × 200 mL). The combined organic layers were washed with brine (300 mL), dried over Na_2_SO_4_, filtered and concentrated under reduced pressure to yield a brown oily crude. The crude was purified by flash column chromatography (gradient elution with hexane:EtOAc 80:20 to 50:50 each containing 0.1% ammonia) to afford the title compound **5** as a clear oil (589 mg, 1.18 mmol, 44%). ^1^H NMR (400 MHz, CDCl_3_) δ 7.44 (d, *J* = 7.3 Hz, 1H), 7.31 (d, *J* = 9.1 Hz, 1H), 7.11 (d, *J* = 8.0 Hz, 1H), 6.68–6.62 (m, 2H), 4.61 (d, *J* = 6.7 Hz, 1H), 3.78 (s, 3H), 3.27 (t, *J* = 12.8 Hz, 1H), 3.20–3.12 (m, 1H), 3.04–2.96 (m, 1H), 2.72–2.58 (m, 5H), 2.55 (d, *J* = 12.0 Hz, 1H), 2.44 (t, *J* = 11.9 Hz, 1H), 1.69–1.52 (m, 4H). ^13^C NMR (101 MHz, CDCl_3_) δ 159.9 (d, *J* = 249.1 Hz), 159.1, 141.2, 135.5, 134.8 (d, *J* = 6.4 Hz), 130.6 (d, *J* = 17.7 Hz), 129.8, 122.1 (d, *J* = 9.5 Hz), 120.8 (d, *J* = 27.4 Hz), 119.4, 116.7, 110.4, 72.4, 60.9, 59.5, 56.2, 55.4, 36.9, 28.5, 27.6, 26.6. ^19^F NMR (376 MHz, CDCl_3_) δ −118.1. HRMS (ESI) calcd for C_21_H_25_Br_2_FNO_2_ [M + H]^+^ 500.0231; found 500.0230.

#### 3.4.5. 3-(4-(4,5-Dibromo-2-fluorophenyl)butyl)-2,3,4,5-tetrahydro-1h-benzo[d]azepine-1,7-diol (Br_2_-OF-NB1, **8**)

To a stirring solution of 3-(4-(4,5-dibromo-2-fluorophenyl)butyl)-7-methoxy-2,3,4,5-tetrahydro-1H-benzo[d]azepin-1-ol (1.0 eq., 589.5 mg, 1.18 mmol) in DCM (16.8 mL) tribromoborane (7.4 eq., 8.7 mL, 8.66 mmol) was added dropwise at −78 °C. The solution was stirred at −78 °C for 10 min and subsequently allowed to come to room temperature. Stirring was continued at room temperature for 3 h. The reaction was quenched with H_2_O, and the pH was adjusted to 7 with NaOH (4 M). The mixture was diluted with H_2_O (200 mL) and extracted with DCM (3 × 200 mL) and ethyl acetate (2 × 200 mL). The combined organic layers were back extracted with brine (500 mL), dried over Na_2_SO_4_, filtered and concentrated under reduced pressure. The crude was purified twice by flash column chromatography (isocratic elution with hexane:EtOAc 20:80 containing 0.1% ammonia) to afford the title compound **6** as a yellow solid (149.1 mg, 0.306 mmol, 26%). ^1^H NMR (400 MHz, CDCl_3_) δ 7.44 (d, *J* = 7.2 Hz, 1H), 7.31 (d, *J* = 9.1 Hz, 1H), 7.03 (d, *J* = 7.8 Hz, 1H), 6.60–6.53 (m, 2H), 4.60 (d, *J* = 6.7 Hz, 1H), 3.29–3.12 (m, 2H), 3.04–2.95 (m, 1H), 2.66–2.52 (m, 6H), 2.44 (t, *J* = 11.9 Hz, 1H), 1.68–1.49 (m, 4H). ^13^C NMR (101 MHz, CDCl_3_) δ 155.2, 141.5, 135.5, 134.8 (d, *J* = 6.1 Hz, 2C), 130.7, 130.1 (2C), 121.0, 120.7, 117.6, 112.5, 72.5, 60.9, 59.5, 56.2, 36.8, 28.5, 27.6, 26.7. ^19^F NMR (376 MHz, CDCl_3_) δ −118.1. HRMS (ESI) calcd for C_20_H_22_Br_2_FNNaO_2_ [M + Na]^+^ 507.9894; found 507.9893.

### 3.5. In Vitro GluN1/2B Competitive Binding Assay

Competitive binding assays were performed as previously reported [23]. Briefly, IC_50_ binding affinity assays were conducted using Wistar rat brain homogenates and HEPES buffer (30 mM HEPES, 110 mM NaCl, 5 mM KCl, 2.5 mM CaCl_2_ and 1.2 mM MgCl_2_). A dilution series of the test ligands was prepared ranging from 30 µM to 300 pM. The cold ligand was displaced with 4.7 nM of either [^3^H]ifenprodil or [^3^H]OF-NB1. Total binding was measured without the cold ligand, and non-specific binding was measured with 100 µM CP101,606 instead of the cold ligand. Each measurement vial contained 0.5 mg/mL of brain homogenate proteins, 20 µL of cold ligand, 10 µL of radioligand and was diluted to 200 µL with HEPES buffer. The vials were incubated at 25 °C and 110 rpm for 1 h. The mixtures were quenched with buffer and filtered through Whatman^®^ GF/C 25 mm filters soaked with 0.05% PEI solution. Filters were washed twice with cold buffer and placed in scintillation vials. Scintillation vials were filled with 2 mL Ultima Gold^TM^ LSC cocktail and measured in a Beckmann LS6500 liquid scintillator counter.

### 3.6. In Vitro σ1R Competitive Binding Assay

The σ1R competitive binding assay was performed in line with previously reported procedures [23]. IC_50_ binding affinity assays were conducted using Wistar rat brain homogenates, and a HEPES buffer (30 mM HEPES, 110 mM NaCl, 5 mM KCl, 2.5 mM CaCl_2_ and 1.2 mM MgCl_2_). Dilution series were prepared with the test cold ligands in a concentration ranging from 30 µM to 300 pM. The cold ligand was displaced with 2.5 nM of (+)-[^3^H]pentazocine. Total binding was measured without the cold ligand, and non-specific binding was measured with 100 µM eliprodil instead of the cold ligand. Each measurement vial contained 0.75 mg/mL of brain homogenate proteins, 20 µL of cold ligand, 10 µL of radioligand and was diluted to 200 µL with HEPES buffer. The vials were incubated at 37 °C and 110 rpm for 2.5 h. The mixtures were quenched with buffer and filtered through Whatman^®^ GF/C 25 mm filters soaked with 0.05% PEI solution. Filters were washed twice with buffer and placed in scintillation vials. Scintillation vials were filled with 2 mL Ultima Gold^TM^ LSC cocktail and measured in Beckmann LS6500 liquid scintillator counter.

### 3.7. In Silico Simulation

#### 3.7.1. Preparation of Co-Crystallized Protein Structure of 3QEL and 6DK1

The crystal structure of NMDA-GluN1b/GluN2B dimer in complex with ifenprodil (PDB ID: 3QEL) and σ1R bound to (+)-pentazocine (PDB ID: 6DK1) were selected for docking simulations. [35,36] The simulations were performed using Molecular Operating Environment (MOE 2019.0101, Chemical Computing Group, ULC, Montreal, QC, Canada, H3A 2R7, 2021) software. After loading the structures in MOE, they were prepared for docking simulations using the default parameters in the ‘QuickPrep Panel’, including the removal of water molecules 4.5 Å away from the ligand pocket, adding hydrogen atoms to the protein structure, adjusting protonation states, and ensuring the energy minimization of the protein structures with an Amber10:EHT force field.

#### 3.7.2. Validation of the Docking Protocol

In order to validate the docking protocol for the two selected targets implemented in the study (described below), the co-crystallized ligands were re-docked in the receptor binding pocket. The ability to reproduce the reported interactions with a minimum root mean square distance (RMSD) value between the co-crystallized pose and docked pose validate the methodology. The RMSD values obtained from the re-docking of the co-crystal ligands of both 3QEL and 6DK1 were 0.257 Å and 0.250 Å, respectively.

#### 3.7.3. Ligands Dataset Curation for Docking Simulations

The ‘builder program’ implemented in MOE was used to model both ifenprodil and OF-NB1, where all possible conformations at the physiological pH were obtained.

#### 3.7.4. Docking Simulations

Docking of the obtained conformations of both ligands (ifenprodil and OF-NB1) was carried out using placement and refinement algorithms of the MOE program. Initial docking of the molecules in the active sites used the ‘Triangle Matcher’ placement method and the ‘London dG’ scoring function. Further postplacement refinement of docking poses was achieved by using the ‘GBVI/WSA dG’ scoring method. The poses with minimum energy were used for visualization of the binding interactions as well as occupancy of the binding site of both receptors. Docking overlays are presented in the Supplementary Material (Section S4, Figures S4 and S5).

## 4. Conclusions

In conclusion, we successfully synthesized and evaluated a novel GluN1/2B radioligand, [^3^H]OF-NB1, for preclinical GluN1/2B ligand development. Its superiority over the current state of the art was demonstrated in a head-to-head comparison by in vitro binding assays, and we showed that systemic errors arise from the use of an unselective radioligand, such as [^3^H]ifenprodil, in GluN1/2B binding affinity screening assays. Our aim is to raise awareness for the need to continuously improve the ligand development toolkit. Due to the high potential of GluN1/2B antagonists exhibiting off-target effects toward σRs and vice versa, we envision that the use of [^3^H]OF-NB1 in GluN2B-targeted drug discovery will facilitate the identification of highly selective candidate drugs with the potential to hold up to expectations in clinical trials.

## Figures and Tables

**Table 1:** Results of in vitro GluN1/2B and σ1R binding affinity testing for CP-101,606 and SA-4503 in nM ± STD.

Radioligand	Test Compound	K_i_ (GluN1/2B)	K_i_ (σ1R)
[^3^H]OF-NB1	SA-4503	>100,000	
	CP-101,606	53 ± 4.3	
[^3^H]Ifenprodil	SA-4503	51 ± 13	
	CP-101,606	37 (16 *)	
[^3^H](+)-Pentazocine	SA-4503		3.8 (4.6 *)
	CP-101,606		94 ± 6

* Reported values in the literature [31,32].

## Data Availability

The data generated and analyzed during our research are not available in any public database or repository but will be shared by the corresponding author upon reasonable request.

## Supplementary Materials

The following supporting information can be downloaded at https://www.mdpi.com/article/10.3390/ph15080960/s1. NMR spectra of compounds 3, 7 and 8, Section S1. Quality control chromatogram and mass spectrum, Section S2, Figures S1 and S2. PET images, Section S3, Figure S3. Docking overlays, Section S4, Figures S4 and S5.
