# Supplementary material for: Development and Validation of [3H]OF-NB1 for Preclinical Assessment of GluN1/2B Candidate Drugs
Source: Pharmaceuticals (Basel). 2022 Aug 2;15(8):960. doi: 10.3390/ph15080960 (PMC9416150; doi:10.3390/ph15080960)
Supplement: Supplementary file 1 [file pharmaceuticals-15-00960-s001.zip › pharmaceuticals-1774310-supplementary.pdf]

Supporting information for

Development and Validation of [^{3}H]OF-NB1 for Preclinical Assessment of GluN1/2B Candidate Drugs

Hazem Ahmed [1,†], Livio Gisler [1,†], Nehal H. Elghazawy [2], Claudia Keller [1], Wolfgang Sippl [2], Steven H. Liang [3], Ahmed Haider [3,*] and Simon M. Ametamey [1,*]

[1] Center for Radiopharmaceutical Sciences ETH-PSI-USZ, Institute of Pharmaceutical Sciences ETH, Vladimir-Prelog-Weg 4, 8093 Zurich, Switzerland

[2] Institute of Pharmacy, Department of Medicinal Chemistry, Martin-Luther-University Halle-Wittenberg, W.-Langenbeck-Str. 4, 06120, Halle, Germany

[3] Division of Nuclear Medicine and Molecular Imaging, Massachusetts General Hospital, and Department of Radiology, Harvard Medical School, Boston, Massachusetts 02114, United States

‡ These authors made equal contributions to the paper.
*Corresponding authors:
Dr. Ahmed Haider; email: Ahmed.Haider@usz.ch
Prof. Dr. Simon M. Ametamey; email: simon.ametamey@pharma.ethz.ch

S1. NMR spectra

S1.1.1 ^{1}H-NMR of compound 3

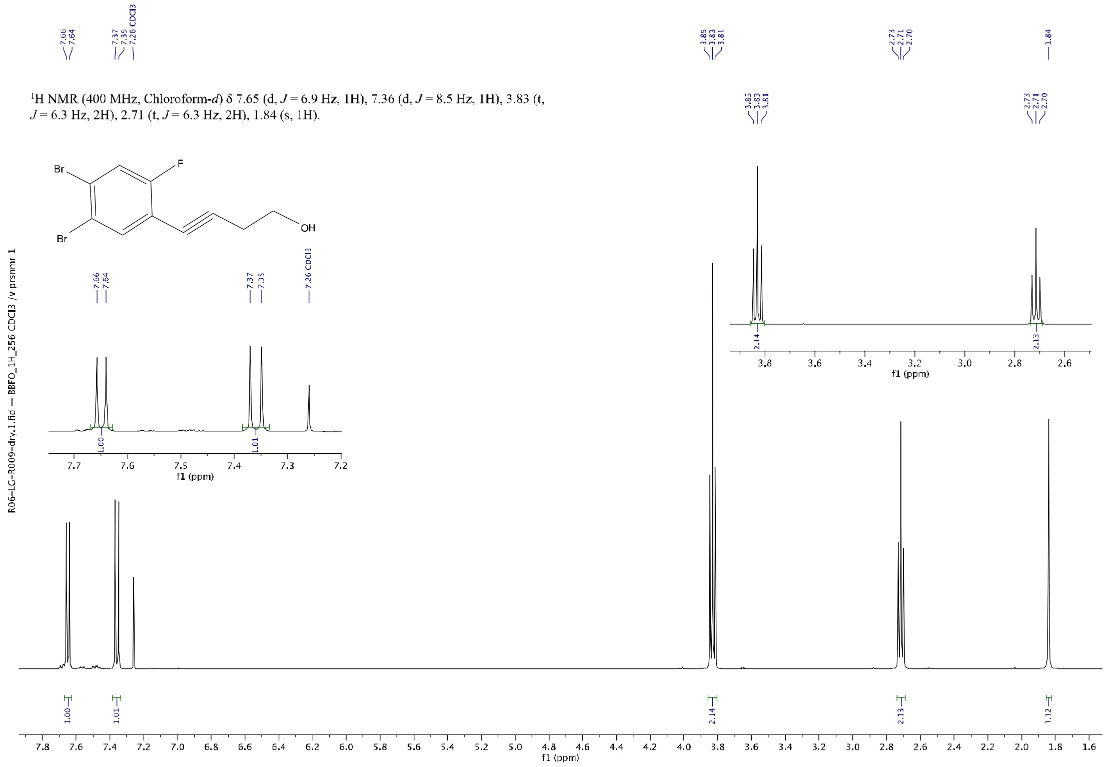

S1.1.2 ^{13}C-NMR of compound 3

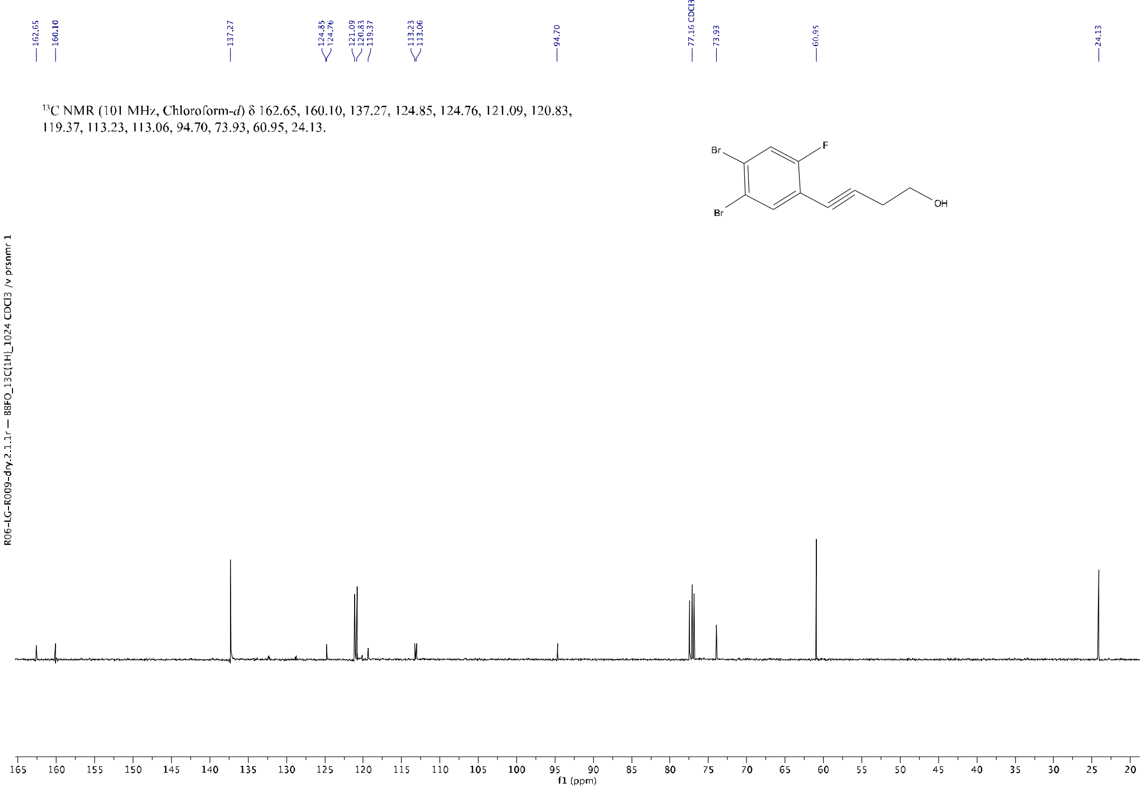

S1.1.3 ^{19}F-NMR of compound 3

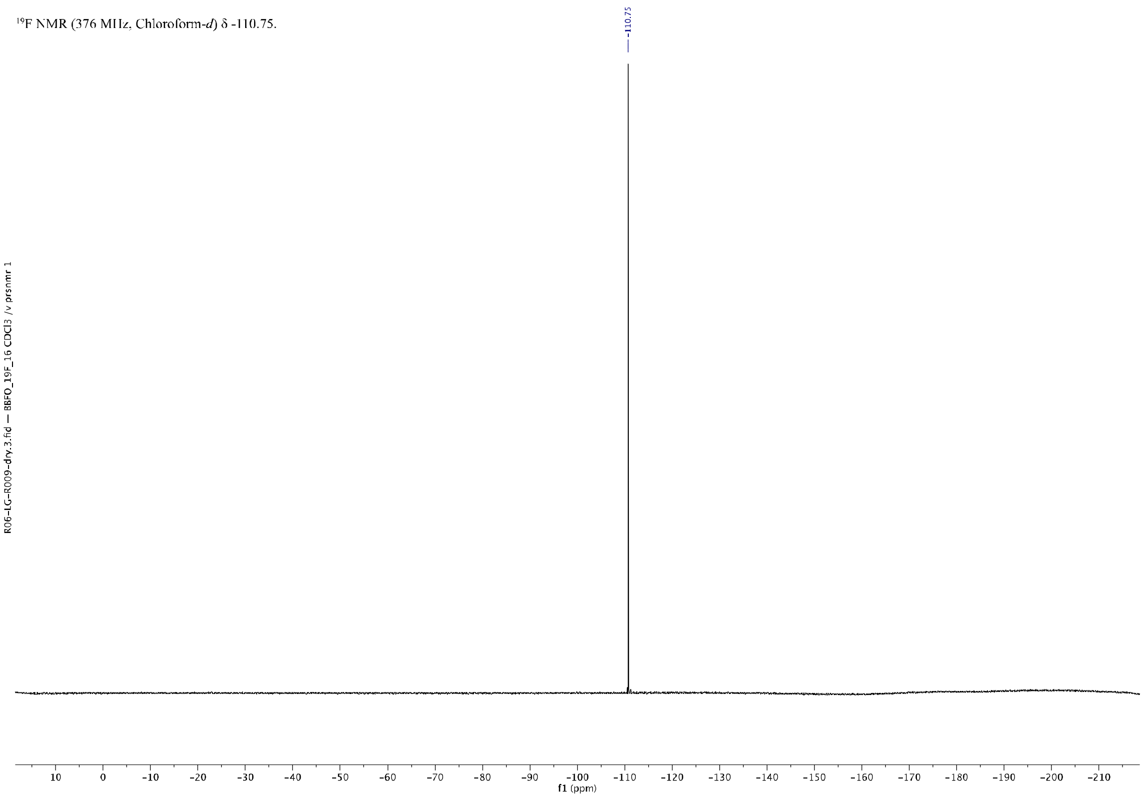

S1.2.1 ^{1}H-NMR of compound 7

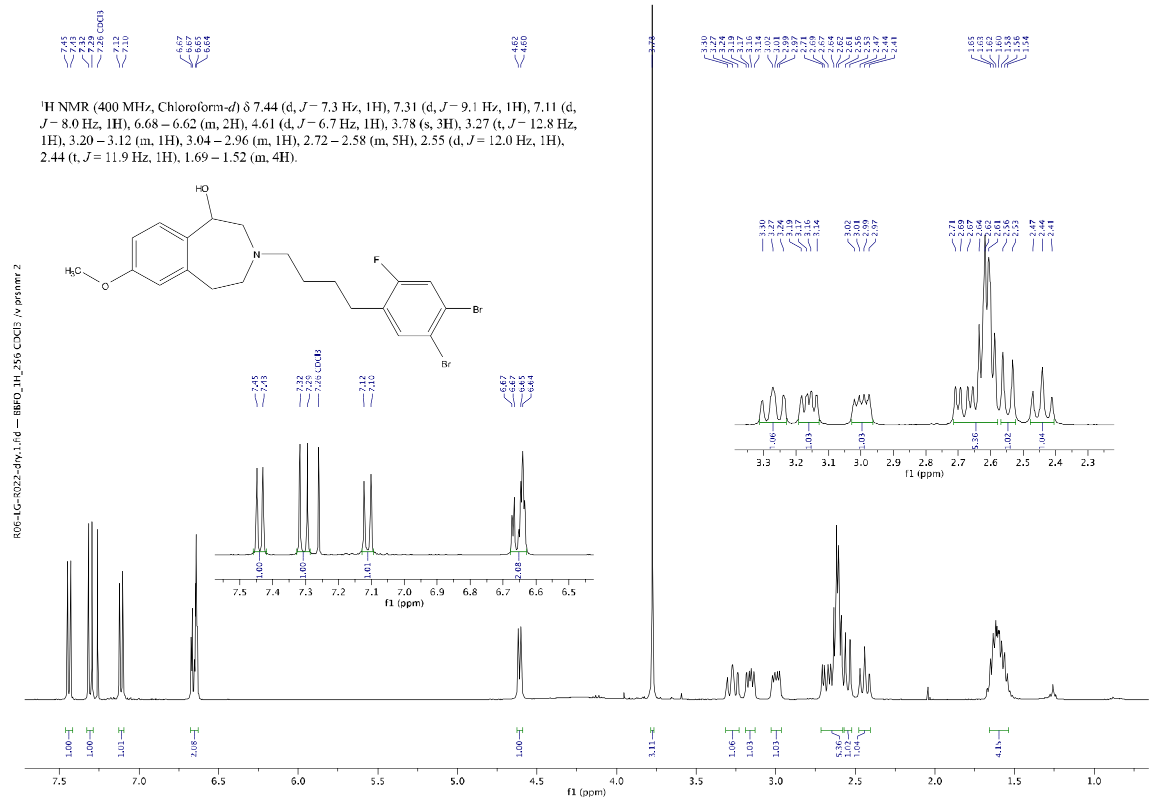

S1.2.2 ^{13}C-NMR of compound 7

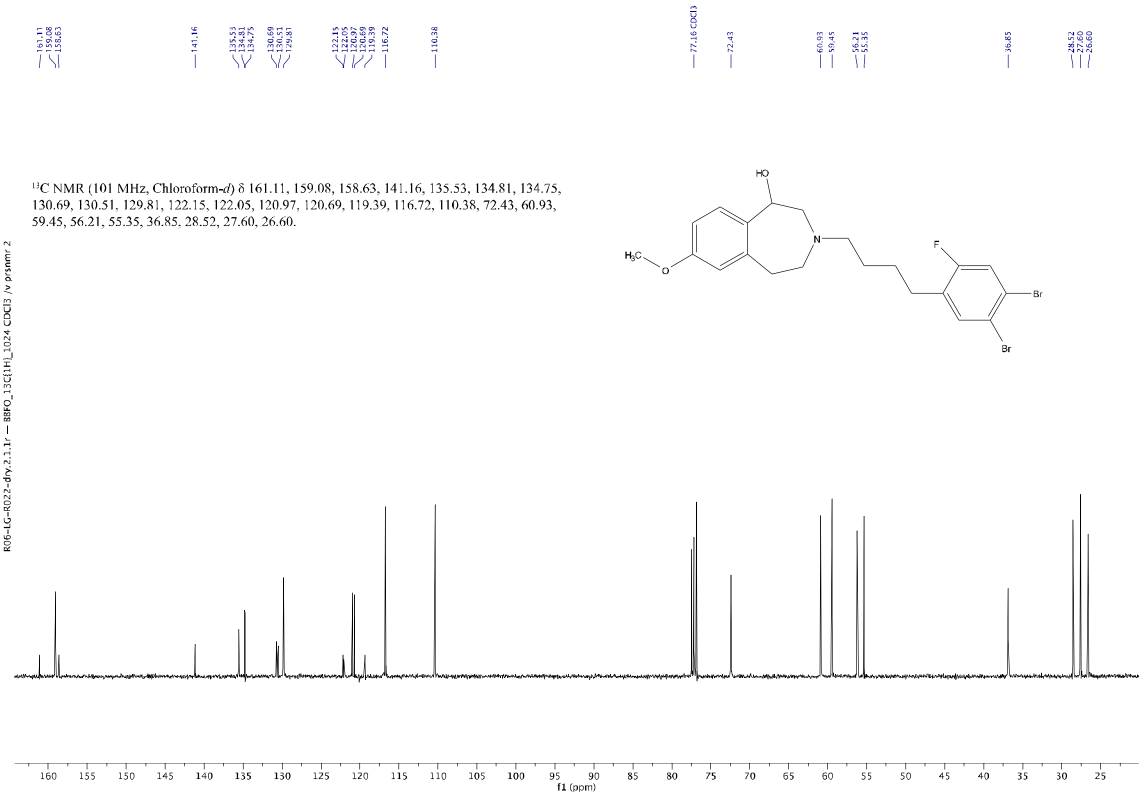

S1.2.3 ^{19}F-NMR of compound 7

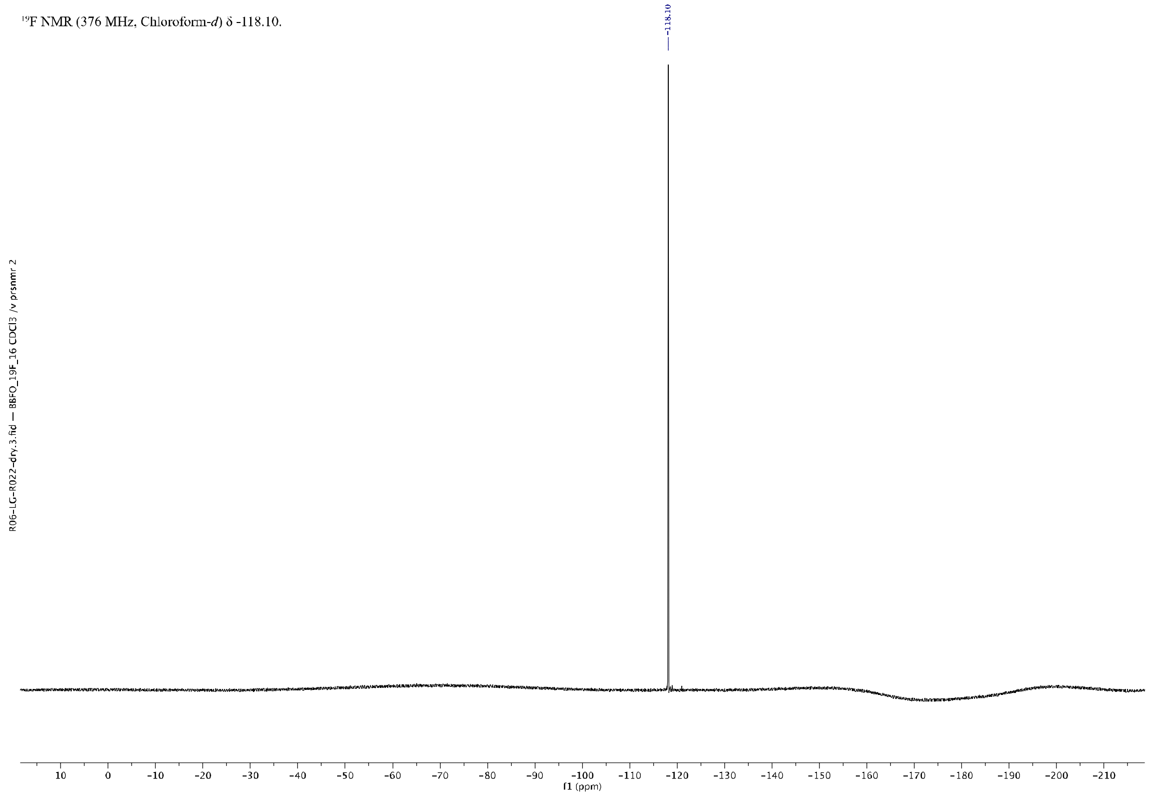

S1.3.1 ¹H-NMR of compound 8

¹H NMR (400 MHz, Chloroform-*d*) δ 7.44 (d, *J* = 7.2 Hz, 1H), 7.31 (d, *J* = 9.1 Hz, 1H), 7.03 (d, *J* = 7.8 Hz, 1H), 6.60 – 6.53 (m, 2H), 4.60 (d, *J* = 6.7 Hz, 1H), 3.29 – 3.12 (m, 2H), 3.04 – 2.95 (m, 1H), 2.66 – 2.52 (m, 6H), 2.44 (t, *J* = 11.9 Hz, 1H), 1.68 – 1.49 (m, 4H).

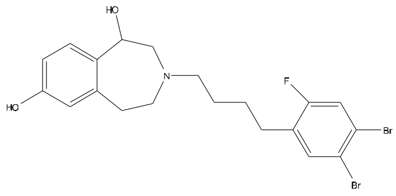

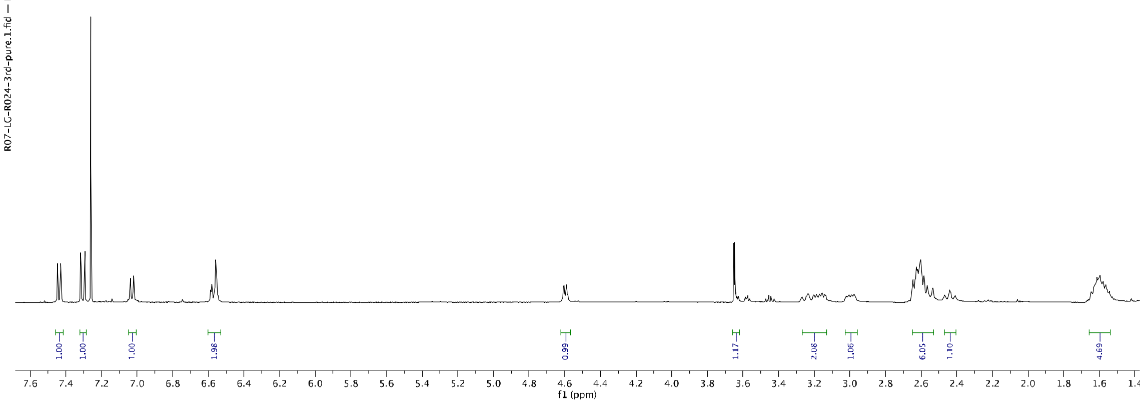

S1.3.2 ^{13}C-NMR of compound 8

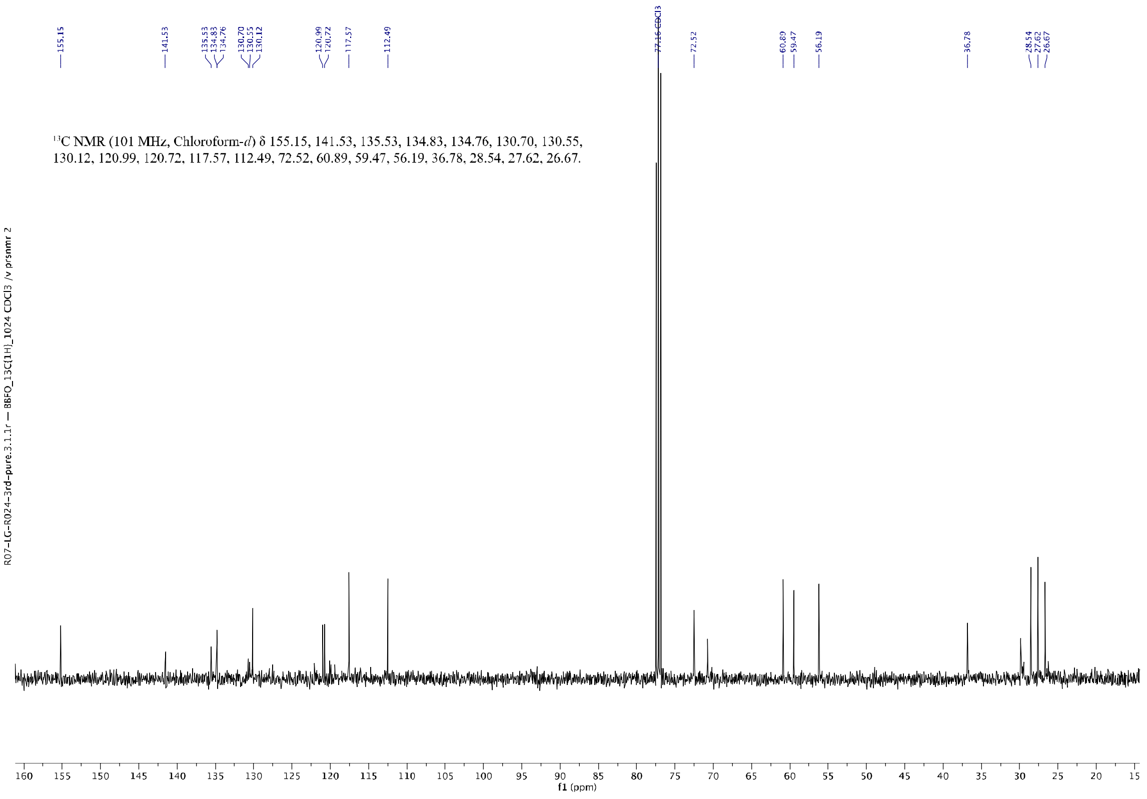

S1.3.3 ^{19}F-NMR of compound 8

^{19}F NMR (376 MHz, Chloroform-*d*) δ -118.10.

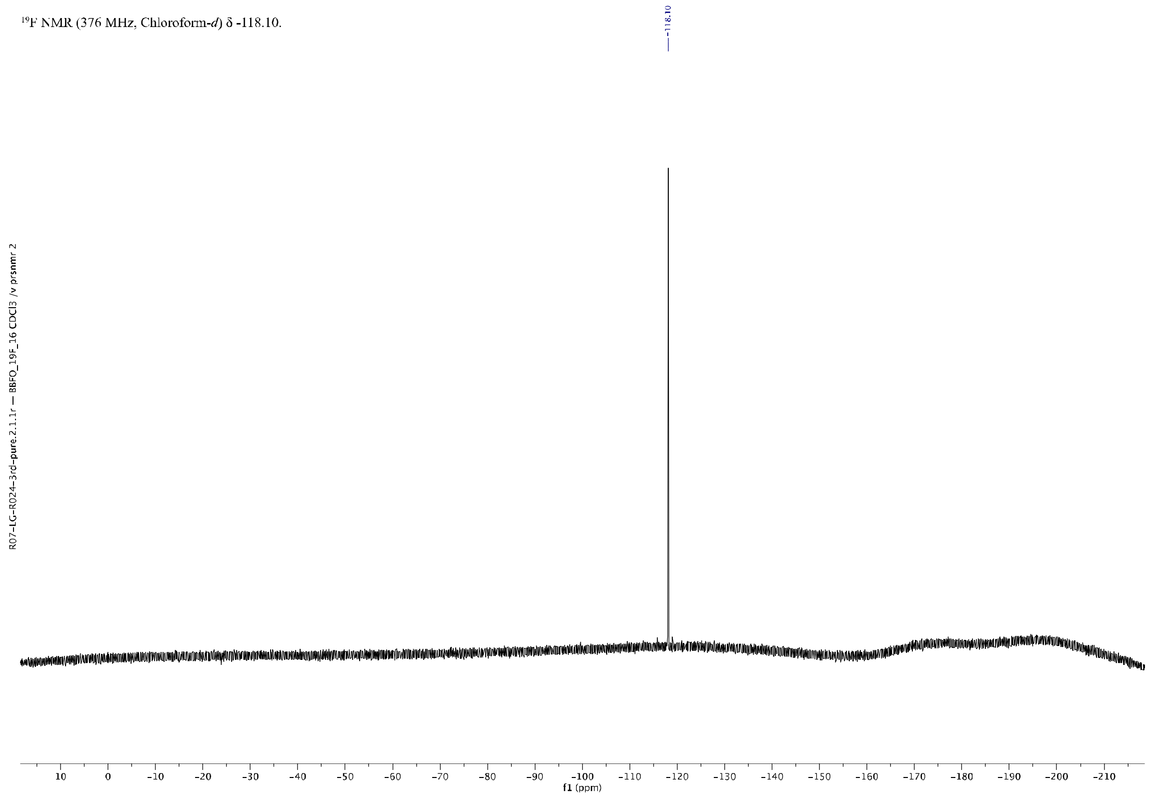

S2. Quality control of [^{3}H]OF-NB1

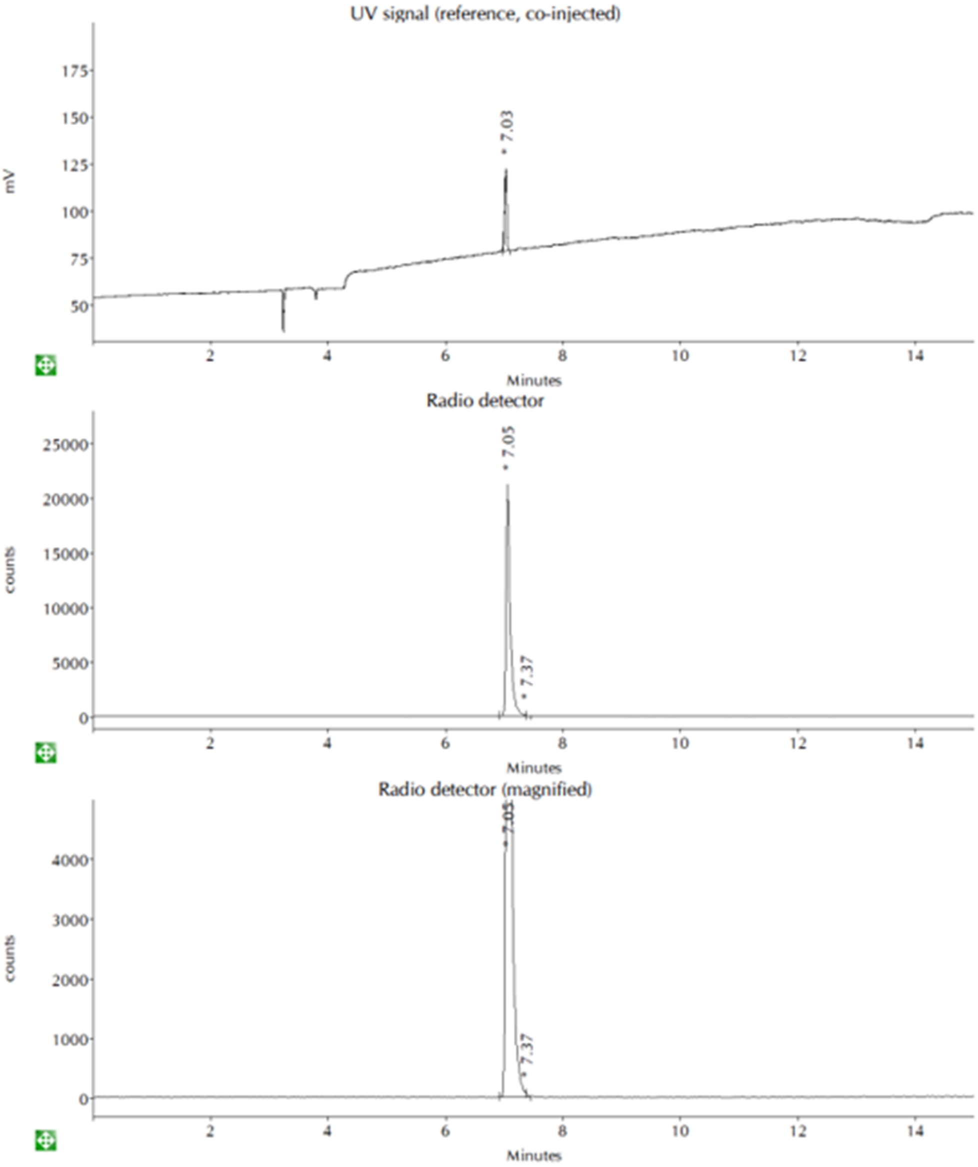

Results radio detector:

#	Peak name	Rt.	Area	% Area
**1**	**OF-NB1**	**7.05**	**104091.88**	**99.88**
2		7.37	120.13	0.12
Sum			104212.00	100.00

Supp. Figure S1. Quality control chromatogram of [^{3}H]OF-NB1 provided by Tritec. The identity of the raditracer by co-injection with OF-NB1. Radiochemical purity was found to be >99%. The sample was injected in a HPLC system equipped with a Waters Sunfire C18 (5 µm), 4.6 x 250 mm HPLC column. The starting mobile phase composition was A: water + 0.05% TFA; B: MeCN + 0.05% TFA. The radiostracer was eluted at a flow rate of 1.0 mL/min using the following conditions; 0 min 10% B; 10 - 14.5 min 95% B; 15 min 10% B.

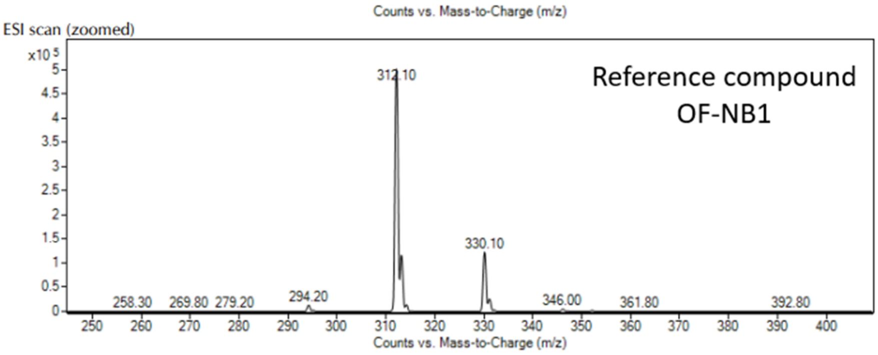

MS peak table of the reference compound

m/z	Abundance
330.1	122710.4
331.1	25942.9
332.1	3583.1
333	314.5
333.9	225.2
335.1	446.8
336	155.8
337.2	95.6

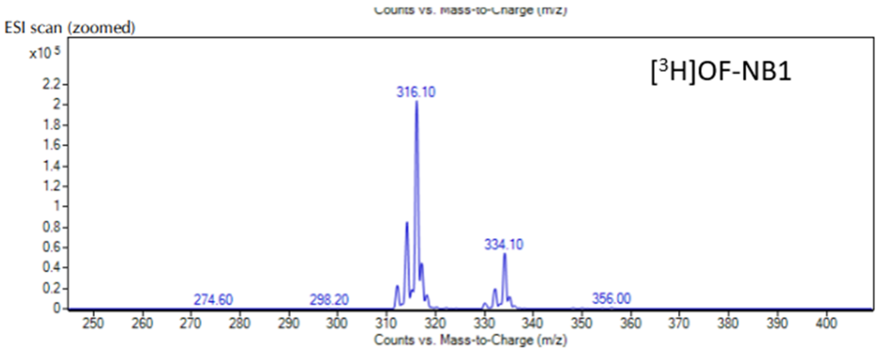

MS peak table of the labeled compound

m/z	Abundance
330	5665.78
331.2	1164.22
332.2	19778.55
333.2	5004.78
334.1	54751.22
335.1	12048.89
336.1	3220.67
337.2	895.89
338.1	402.67
339.1	178.67
340	0
341	157.67
342.2	109.89

Supp. Figure S2. Mass spectrum of the reference compound and [^{3}H]OF-NB1 after measurement on a LC-MS as provided by Tritec. The data was recorded on an Agilent LC-MS 6120 (single quadrupole) after eluting from an Agilent Zorbax Eclipse Plus C18 column (1.8 µm); 2.1 x 50 mm. The starting mobile phase composition was A: water + 0.1% TFA; B: MeCN + 0.1% TFA. The radiostracer was eluted at a flow rate of 0.6 mL/min using the following conditions; 0 min 5% B; 0.2 min 5% B; 4 min 95% B.

S3. PET imaging of a Wistar rat using [^{18}F]fluspidine

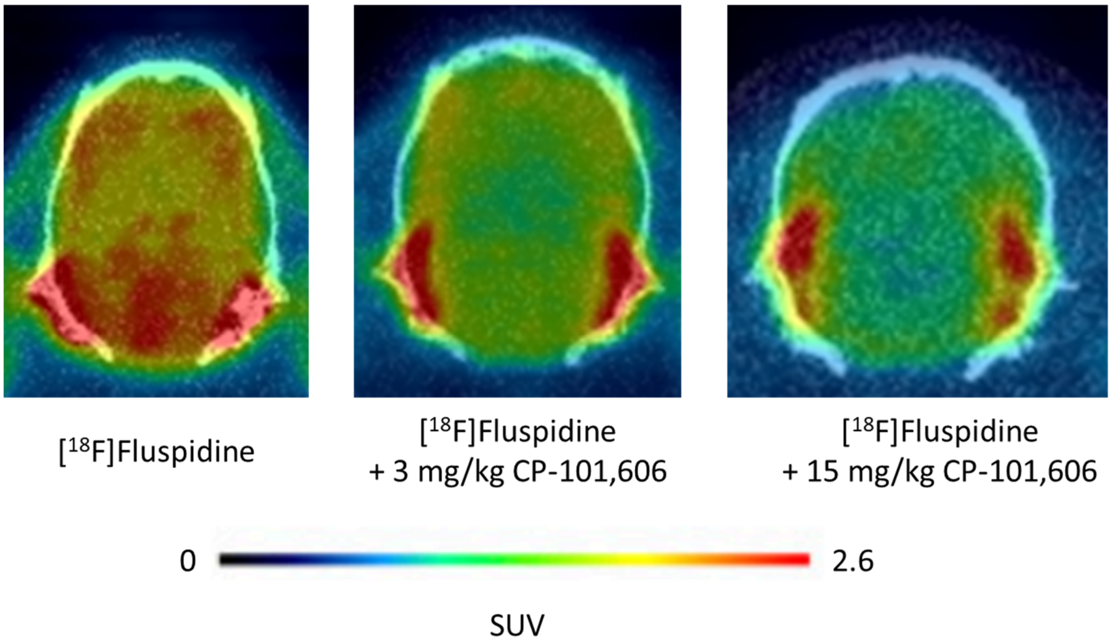

Supp. Figure S3. Coronal PET images of [^{18}F]fluspidine averaged over 90 min in Wistar rats. The brain images are shown for rats under baseline and blockade conditions (3 and 15 mg of CP-101,606 per kilogram) superimposed on the respecive recorded CT image. The colour indicates the minimum and maximum standard uptake values (SUVs). The SUV is defined as the accumulated radioactivity [Bq] per tissue [g] / injected dose per body weight.

S4. Docking overlay

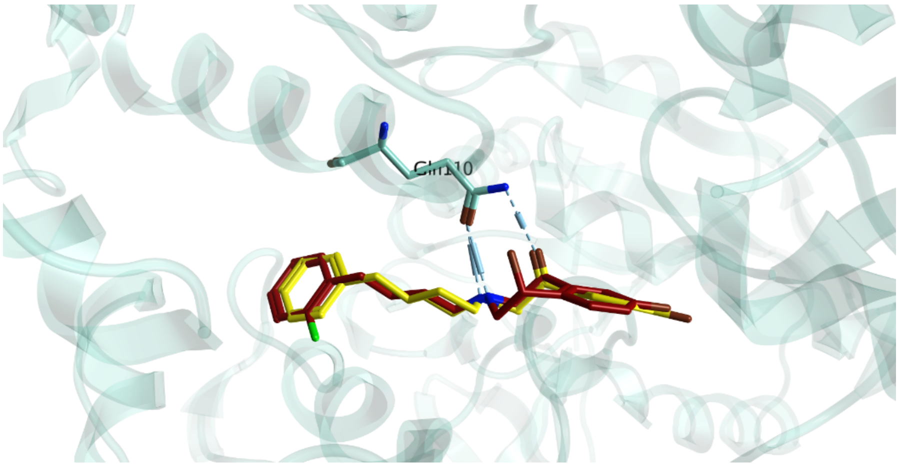

Supp. Figure S4. Docking overlay of ifenprodil and OF-NB1 againist the GluN1b/2B receptor (3QEL).

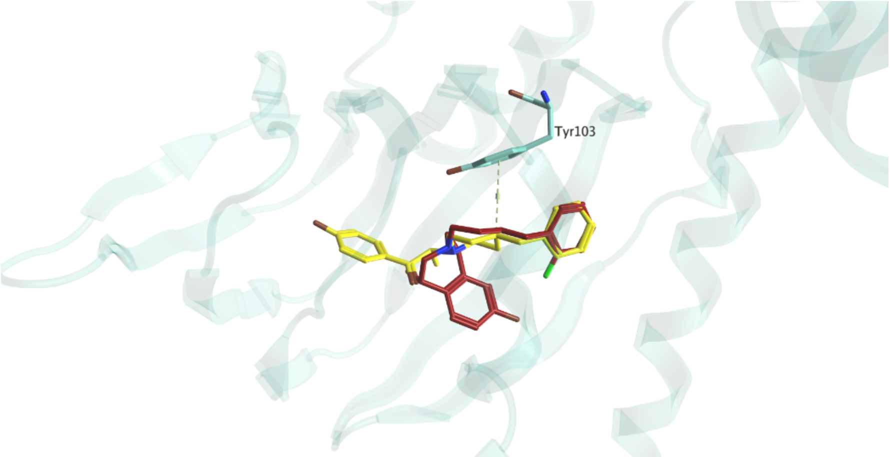

Supp. Figure S5. Docking overlay of ifenprodil and OF-NB1 againist the σ1 receptor (6DK1).
